# Giant juvenile fibroadenoma in an adolescent female: A case report

**DOI:** 10.1002/ccr3.3466

**Published:** 2020-10-31

**Authors:** Suman Baral, Milan Gyawali, Neeraj Thapa, Raj Kumar Chhetri, Prahar Dahal

**Affiliations:** ^1^ Department of Surgery Lumbini Medical College and Teaching Hospital Tansen Nepal; ^2^ Department of Pathology Lumbini Medical College and Teaching Hospital Tansen Nepal

**Keywords:** giant juvenile fibroadenoma, phyllodes tumor

## Abstract

Giant juvenile fibroadenoma in adolescents should be dealt with utmost caution as this may be associated with anxiety, fear, and emotional factors. The treatment should aim for preserving the normal contour of the breast along with appealing scar.

## Graphical abstract

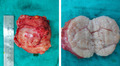

## 1 INTRODUCTION

Giant juvenile fibroadenoma is one of the rarer clinical findings in adolescent females which needs to be differentiated from phyllodes tumor, physiological hypertrophy, and other inflammatory lesions like breast abscess. Treatment includes removal of tumor with maintenance of normal breast contour.

Fibroadenoma of the breast is one of the common lesions in adolescent females with over all incidence of 2.2%.^1^, ^2^ Simple fibroadenoma is the most common entity which includes 70%‐90% of the cases while giant juvenile fibroadenoma is the rare variant.^3^ A juvenile fibroadenoma is considered giant when the size exceeds >5 cm, weighs more than 500 gm, or replaces at least 80% of the breast.^4^ Differentials include phyllodes tumor, physiological hypertrophy, and other inflammatory lesions like breast abscess.^5^ Due to propensity of rapidly growing, this tumor might create self‐consciousness, discomfort, and anxiety that just impacts the psychological and emotional aspects of the individual.^6^ We articulate a clinical case of an adolescent female who presented with rapidly growing juvenile fibroadenoma who was managed with enucleation of the tumor at our clinical setting.

## 2 CASE REPORT

**FIGURE 1:**
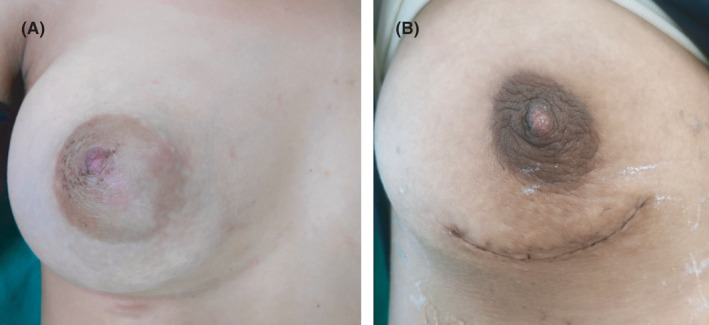
A, showing the lump in the right breast involving central area with right upper and lower quadrant of the breast. B, showing the postoperative inframammary scar during follow‐up with normal contour of the right breast

**FIGURE 2:**
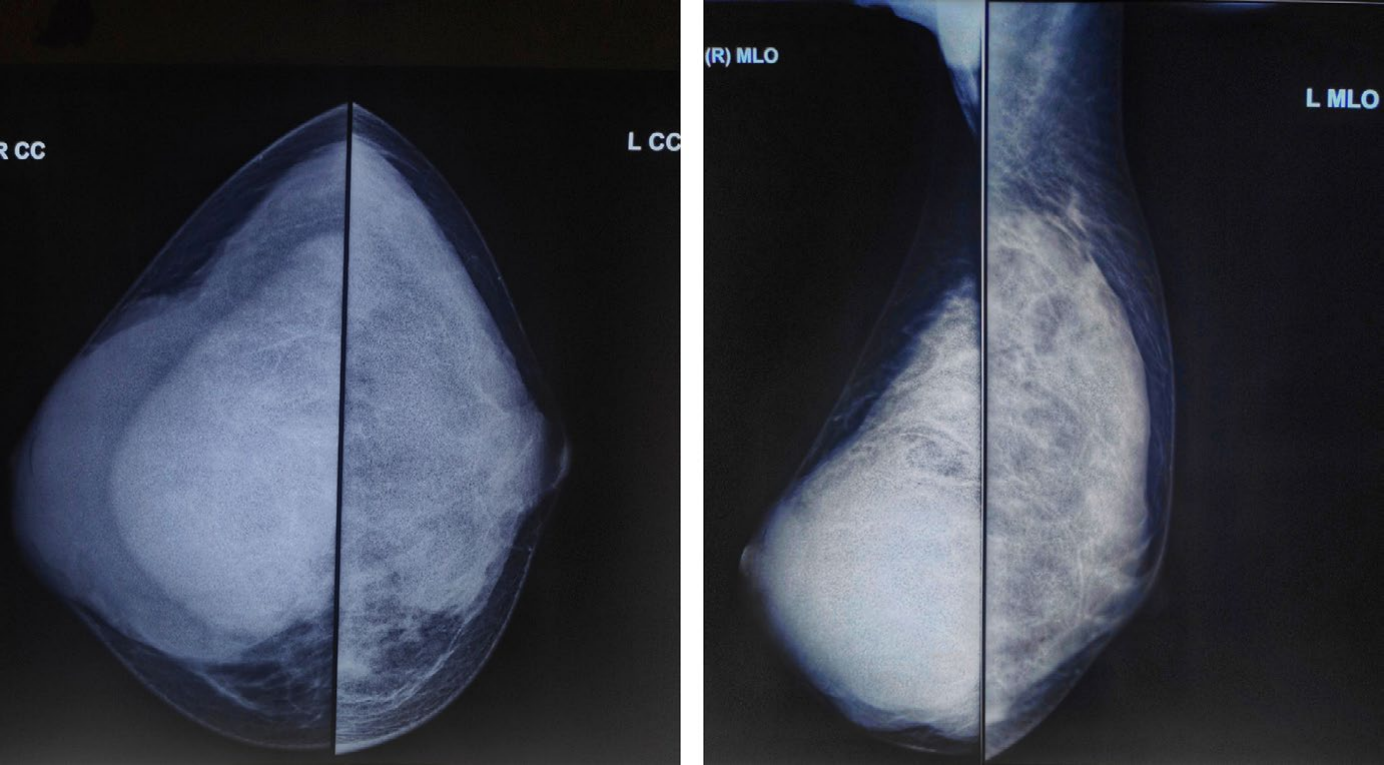
Showing the mammogram of bilateral breast showing craniocaudal (CC) and mediolateral oblique (MLO) view showing dense fibro‐glandular parenchyma of bilateral breast with well‐defined large oval‐shaped equal to low‐density lesion with lobulated margins in the peri‐areolar region of right breast (BIRADS‐3)

**FIGURE 3:**
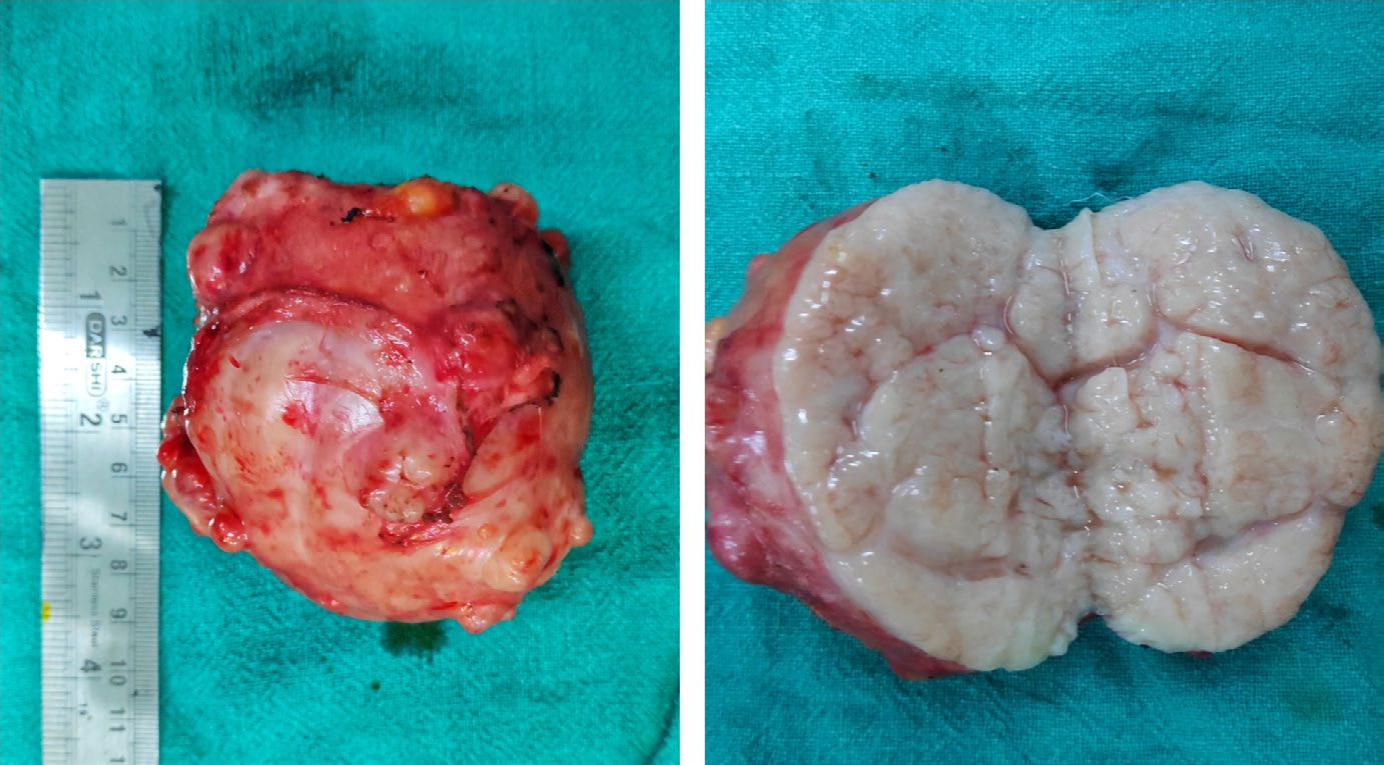
Showing the excisional biopsy of the right breast lump measuring approximately (9 × 7 × 6.5) cm, firm, well‐circumscribed, ovoid creamy white with bosselated surface. Cut surface shows slit‐like spaces along with two cystic spaces and lobulations bulging over the cut surface

**FIGURE 4:**
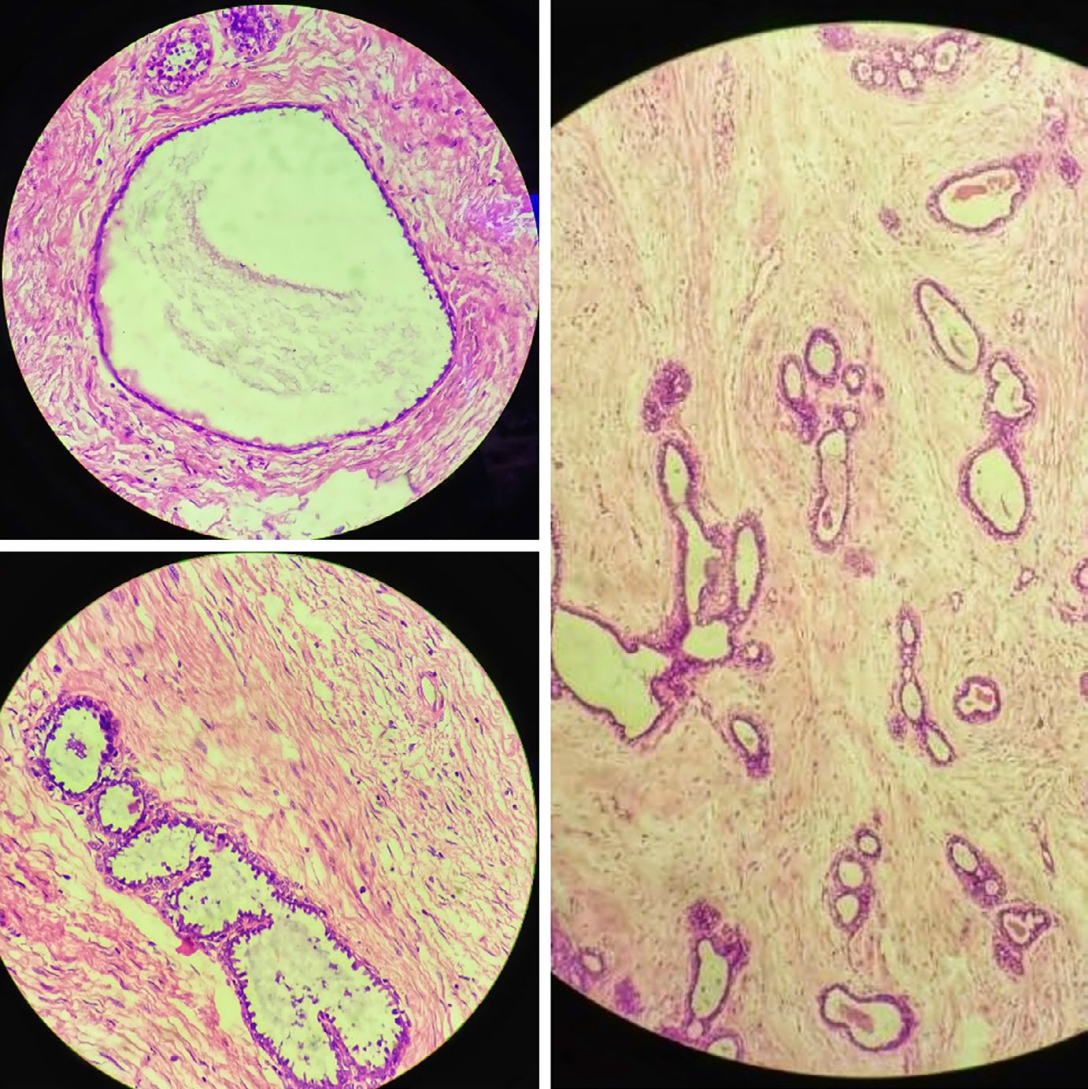
Showing the cyst formation more than 3 mm with apocrine changes—cells with abundant eosinophilic cytoplasm and apical snouts along with glandular epithelium with intact myoepithelial cell layers suggestive of complex fibroadenoma

An 18‐year‐old unmarried lady presented to surgical outpatient department with complaints of lump in the right breast for 6 months. The lump was gradual in onset, freely mobile, initially a size of a marble, gradually which increased to the present size throughout the duration of time. The mass was not associated with pain or skin color changes or any abnormal discharge per nipple. There was no any history of trauma, fever, cough, or difficulty in breathing. There was no history of similar illness among the family members or first‐degree relatives, chest irradiation, or any use of oral contraceptive pills. Her menarche was at 13 years with regular menstrual cycle. Vitals were stable. All the laboratory and biochemical parameters along with systemic examination were within the normal limit. Right breast examination revealed a freely mobile mass involving central area of the breast along with right upper and lower quadrant approximately the size of 10 cm × 9 cm with no adherence to the skin or fixity to the posterior wall; however, overlying skin was slightly tensed with distended superficial veins (Figure 1A). It was nontender, well circumscribed, and firm in consistency. Nipple appeared slightly fissured. Axillary lymph nodes were not palpable. Left breast examination was normal. Ultrasonography of right breast showed inhomogeneous solid mass measuring 85.9 × 74.6 mm with posterior acoustic enhancement in right breast suggestive of fibroadenoma. Pathological examination with FNAC was not possible due to reluctance of the patient. Mammography bilateral breast showed dense fibro‐glandular parenchyma along with well‐defined, large oval‐shaped equal to low‐density lesion with lobulated margin in the peri‐areolar region of the right breast (Figure 2). The patient was taken for surgery, and enucleation of the tumor was done with inframammary incision along the right breast. Intraoperatively, the tumor was 9 cm × 7 cm in size, firm, ovoid, well circumscribed with bosselated surface with nodularity. Cut section of the mass showed many slit‐like spaces with gray white in color (Figure 3). Postoperative event remained uneventful. The patient got discharged on 4th postoperative day. Histopathology was consistent with complex juvenile fibroadenoma (Figure 4).

Follow‐up clinical examination showed well‐healed wound with scar as seen in Figure 1B.

## 3 DISCUSSION

Fibroadenomas are the most commonly treated breast lesions in adolescence age groups which accounts for 44%‐94% of total biopsied specimens.^4^ These lesions have been classified as simple fibroadenoma which is the most common variant, giant juvenile fibroadenoma, and multicentric fibroadenoma.^7^ It is termed juvenile if it occurs among adolescents of 10‐18 years of age.^8^ Giant juvenile fibroadenoma is a rare encapsulated variant which is defined as size more than 5 cm, weighs more than 500 gm, or displaces at least four fifth area of the breast. The overall incidence of giant fibroadenoma is approximately 0.5%‐2% of fibroadenomas and is the most common cause of unilateral macromastia in younger age groups.^3^

The etiology of fibroadenoma is unclear. Hormonal theories exist which implicates excess estrogen stimulation, increased estrogen receptors, or decreased estrogen antagonist activity in the breast.^9^ Differentials for giant juvenile fibroadenoma include phyllodes tumor and virginal hypertrophy. Giant lesions have potential to disfigure the breast; however, the chances of malignancy are very low. Phyllodes range from benign to malignancy. Virginal hypertrophy is unequal growth of breast buds on one side related to stimulation of hormone. Histologically, phyllodes can be differentiated from giant fibroadenoma by the presence of leaf‐like projections and stromal cell atypia while virginal hypertrophy lacks lobule formation along with presence of abundant connective tissue with ductal proliferation.^2^ Two variants of fibroadenoma have been described by Dupont in his study from Nashville as simple and complex fibroadenoma. Complex variant shows presence of foci of cysts, sclerosing adenosis, epithelial calcifications, and papillary apocrine metaplasia and has a higher future risk of malignancy.^10^

Initially, it is difficult to diagnose giant fibroadenoma with physical examination because of the converging clinical presentation of large mass leading to asymmetrical breasts, skin changes, or deviation of nipple areola complex. Radiological investigations include ultrasonography (USG) and mammography for routine imaging along with magnetic resonance imaging (MRI) in selected cases; however, the definite diagnosis is difficult to be made upon the radiology alone. On USG, fibroadenomas appear as a well‐circumscribed round or oval solid mass, with weak internal echoes in a uniform distribution and intermediate acoustic attenuation; however, the accuracy of diagnosis is around 78.8% in the case series by Smith et al, where the total sample size was 447 with age ≤25 years of age.^11^, ^12^ The use of mammograms in young females has widely been documented to be of limited value due to increased breast density, and utility is limited due to poor image quality in younger patients as well as the extremely low risk of malignancy.^13^, ^14^ Preoperative cytology( FNAC) plays a key role for the diagnosis and to come to a diagnosis; however, its role for differentiating giant fibroadenoma and borderline phyllodes has been difficult due to overlapping of some of their microscopic morphological features.^15^ Also, the minimal sample tissue in needle biopsy may yield uncertain results that mandates excisional biopsy as the last resort for the diagnoses of giant fibroadenoma. Core needle biopsy, in turn, may be more appropriate than FNAC for the preoperative diagnosis which incorporates large amount of sample tissue and diagnostic capabilities are high; however, adolescent patients might not be compliant for the procedure due to intolerability along with psychological and emotional aspects.^16^

Treatment of giant fibroadenoma seems little challenging in the view to pursue and achieve adequate resection margin along with preservation of the maximum amount of physiological breast tissue in the adolescence age. Potential treatment options include simple excision with cosmetically appealing incisions in inframammary fold or peri‐areolar region, reduction mammoplasty, and in some cases mastectomy with reconstruction.^17^ However, we mainly focus on simple excision of the lesion wherever applicable especially in adolescent age groups. Hille‐Betz et al^17^ suggested simple excision of the mass using an inframammary or peri‐areolar approach without reconstructive plasty that showed good cosmetics result along with postoperative outcomes while on follow‐up in their retrospective study of 13 patients where eight had diagnosed fibroadenoma. Park and colleagues assessed nine patients among which seven underwent reduction mammoplasty which was directed to reduce the amount of extra skin and immediately reestablish breast asymmetry; however, this approach results in longer, and often more conspicuous, scarring as well as the typical risks associated with reduction mammaplasty/mastopexy.^17^, ^18^ Narayansingh and colleagues termed the Saw Tooth operation for giant fibroadenoma which is based on the principle that it is better to incise the lump rather than the patient, recognizing that it is benign disease. A small circum‐areolar incision is made, and the tumor is removed by making alternate incisions along the medial and lateral or the superior and inferior surfaces of the lump once this has been freed by blunt finger dissection.^19^ Whatever the technique be applied, the main aim of the surgery should be to provide better cosmetics result, patient satisfaction, and preservation of as much breast as possible especially with the younger age group of patients. In our case, the patient had full satisfaction along with regain of normal breast contour on follow‐up.

## 4 CONCLUSION

Giant fibroadenoma of the breast is one of the rarest findings in adolescents. Simple excision of the lump with inframammary approach can be adopted for the better cosmesis and gratifying postoperative outcome.

## CONFLICT OF INTEREST

None declared.

## AUTHOR CONTRIBUTIONS

SB: wrote the article. MG and RKC: shared in the discussion. NT and PD: revised the manuscript.

## ETHICAL APPROVAL

Written informed consent was obtained from the patient for the publication of the text and images. Ethical approval was not mandatory for publication of case reports as per the institutional policy.

## Data Availability

Data sharing is not applicable to this article as no datasets were generated or analyzed during the current study.
